# Computing the protein binding sites

**DOI:** 10.1186/1471-2105-13-S10-S2

**Published:** 2012-06-25

**Authors:** Fei Guo, Lusheng Wang

**Affiliations:** 1 School of Computer Science and Technology, Shandong University, Jinan 250101, Shandong, China; 2 Department of Computer Science, City University of Hong Kong, 83 Tat Chee Avenue, Kowloon, Hong Kong

## Abstract

**Background:**

Identifying the location of binding sites on proteins is of fundamental importance for a wide range of applications including molecular docking, de novo drug design, structure identification and comparison of functional sites. Structural genomic projects are beginning to produce protein structures with unknown functions. Therefore, efficient methods are required if all these structures are to be properly annotated. Lots of methods for finding binding sites involve 3D structure comparison. Here we design a method to find protein binding sites by direct comparison of protein 3D structures.

**Results:**

We have developed an efficient heuristic approach for finding similar binding sites from the surface of given proteins. Our approach consists of three steps: local sequence alignment, protein surface detection, and 3D structures comparison. We implement the algorithm and produce a software package that works well in practice. When comparing a complete protein with all complete protein structures in the PDB database, experiments show that the average recall value of our approach is 82% and the average precision value of our approach is also significantly better than the existing approaches.

**Conclusions:**

Our program has much higher recall values than those existing programs. Experiments show that all the existing approaches have recall values less than 50%. This implies that more than 50% of real binding sites cannot be reported by those existing approaches. The software package is available at http://sites.google.com/site/guofeics/bsfinder.

## Background

Identifying the location of binding sites on proteins is of fundamental importance for a wide range of applications including molecular docking, de novo drug design, structure identification and comparison of functional sites. Structural genomic projects are beginning to produce protein structures with unknown functions. Therefore, efficient methods are required if all these structures are to be properly annotated.

Many methods have been proposed for identifying the location of binding sites on proteins. Laurie and Jackson give an energy-based method for the prediction of protein-ligand binding sites [1]. Bradford and Westhead combine a support vector machine (SVM) approach with surface patch analysis to predict protein-protein binding sites [2]. Chen *et al. *develop a tool, 3D-partner, for inferring interacting partners and binding models [3]. 3D-partner first utilizes IMPALA to identify homologous structures (templates) of a query protein sequence from heterodimer profile library. The sequence profiles of those templates are then used to search interacting candidates of the query from protein sequence databases by PSI-BLAST. Lo *et al. *develop a method for predicting helix-helix interaction from residue contacts in membrane proteins [4]. They first predict contact residues from sequences. Their relationships are further predicted in the second step via statistical analysis on contact propensities and sequence and structural information. Li *et al. *propose an approach for finding binding sites for groups of proteins [5]. It contains the following steps: finding protein groups as bicliques of protein-protein interaction networks (PPI), identifying conserved motifs, and searching domain-domain interaction databases. Liu *et al. *extend the method of Li *et al. *in [5] and consider comparing 3D local structures [6]. Guo and Wang identify the binding sites by finding two similar 3D substructures [7].

SiteEngine is a method that recognizes the regions on the surface of one protein that are similar to the binding sites of another. It uses geometric hashing triangles to transfer the input sites into the recognized region [8]. SuMo is a system for finding similarities in arbitrary 3D structures or substructures of proteins. It is based on a unique representation of macromolecules using selected triples of chemical groups [9]. The web server pdbFun analyzes the structure and function of proteins at the residue level [10]. When comparing a complete protein with all complete protein structures in the PDB database, experiments show that all the existing approaches have recall values less than 50% implying that more than 50% of real binding sites cannot be reported by those existing approaches.

In this paper, we design a method to recognize regions of binding sites on the proteins. It consists of three steps: local sequence alignment, protein surface detection, and 3D structures comparison. Experiments show that the average recall value of our approach is 82% and the average precision value of our approach is also significantly better than the existing approaches.

## Methods

Given two complete protein structures, our task is to find the binding sites on the given proteins. Our method contains three steps. Step 1, we do local sequence alignment at the atom level to get the alignments of conserved regions. These alignments of conserved regions may contain some gaps. Step 2, among the conserved regions obtained in Step 1, we use the 3D structure information to identify the surface segments. Step 3, for any pair of the surface segments identified in Step 2, we compute a rigid transformation to compare the similarity of the substructures in 3D space and output the qualified pairs as binding sites.

### Step 1: Local sequence alignment

**Figure 1:**
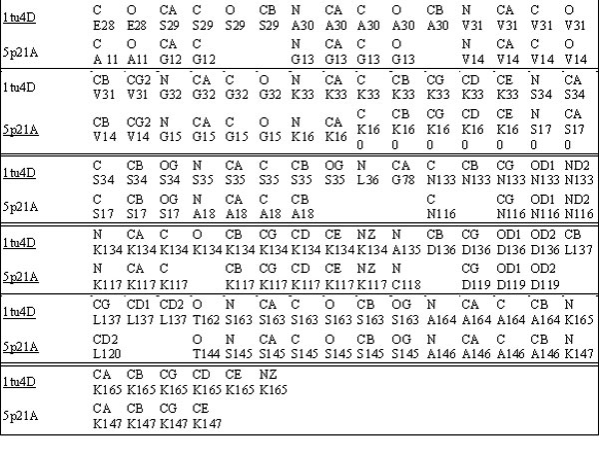
**The binding sites on 1TU4D and 5P21A**.

In PDB format files, each residue (amino acid) is represented in the traditional order of atom records N, CA, C, O, followed by the side chain atoms (CB, CG1, CG2 ...) in order first of increasing remoteness, and then branch. The whole protein sequence of residues can be translated into a sequence of atoms based on this representation. The sequences of binding sites on the proteins are usually conserved at the atom level. When looking at the SitesBase [11], we can see that the pair of binding sites form a conserved region that are well aligned at the atom level, where atoms of the same types are matched and all the unmatched atoms correspond to gaps. Figure 1 is the result of SitesBase for proteins 1TU4D and 5P21A.

We use the standard Smith-Waterman's local alignment algorithm [12] to find the conserved segments, where a matched pair of atoms of the same type has a score 1, a mismatched pair of atoms of different types has a score -∞, a mismatch between an atom and a space has a score -2. The local alignment algorithm can return a set of conserved segments in the alignment of the protein sequences of atoms.

We have done many experiments and found that the set of conserved segments output by the local sequence alignment algorithm always contains the pairs of binding sites in the SitesBase. The only problem is that the local sequence alignment algorithm outputs too many matched atoms. Next, we will further reduce the matched atoms. After obtaining the set of conserved segments from the local sequence alignment, we focus on the columns with identical pairs of atoms and ignore the rest of columns in the following steps.

### Step 2: Identifying surface segments

**Figure 2:**
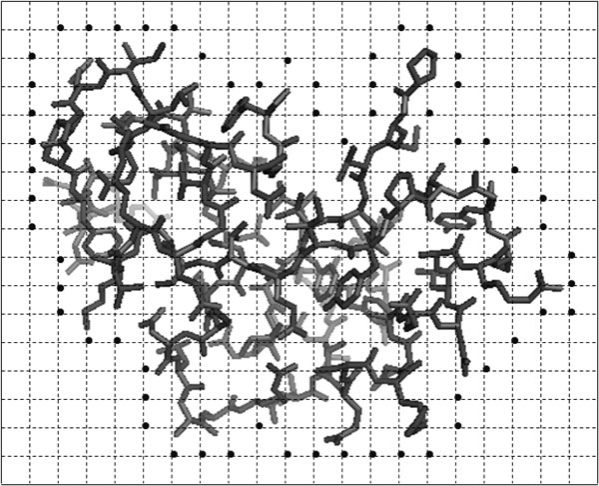
**The surface grid points are indicated by the dark points**.

Inspired by the work in [13], we propose the following method to find surface segment of proteins. First, the protein is projected onto 3D grid in the Euclidean space. For the grid, we use a step size of 1Å. Second, grid points are marked as *interior*, *surface *or *empty*. A grid point is marked as *protein *if the point is within 2Å distance of an atom in the protein. A grid point is marked as *empty *if it is not *protein *point. A grid point is marked as *interior *if all its six neighbor grid points are *protein *points. A grid point is marked as *surface *if at least one of its six neighbor grid points is not *protein *point. An atom in the protein is a *surface *atom if it is within distance 1.5Å of a *surface *point. Figure 2 gives an example, where the dark grid points are surface points.

For a conserved segment output by the local sequence alignment algorithm, we consider all its subsegments containing at least 15 matched pairs of atoms. For such a subsegment, if both sequences on this subsegment have at least 2/3 atoms as the surface atoms, we treat such a subsegment as a candidate binding site for further processing in the next step.

### Step 3: Computing rigid transformations to match candidate binding sites

For any candidate binding sites obtained from Step 2, we will further test if the pair of 3D substructures can match well on such a site. Precisely, we can find the set of subsegments in a given segment with alignment  A using the following rule: there exists a rigid transformation such that the distance between each pair of atoms in the same column of the subsegment is at most *d*, where *d *is a parameter given by the user. A rigid transformation is a transformation for protein 3D structure in the 3D space that preserves distances between any pair of points in the structure of protein. This requires us to solve the following protein 3D structure matching problem:

**Input: **A segment with sequence alignment  A of two proteins, where each position in the alignment has two identical atoms, the 3D coordinate of each atom in the alignment, and a threshold *d*.

**Goal: **Find a set of subsegments with alignment  A such that for each output subsegment the Euclidean distance between each pair of atoms in the same column is at most *d*.

The protein 3D structure matching problem can be solved in several ways. Here we use the method in [14] which is a faster version of the method in [15] to solve the problem. The method in [14] can compute a rigid transformation such that the distance between each matched pair of atoms is at most (1+*∈*)*d*, where *∈ *= 0.1 is a parameter to control the precision of the transformation. This is just an approximate rigid transformation, and it is good enough in practice.

### Testing the overlap of the proteins in 3D space

When computing the rigid transformation, we also require that the proteins do not overlap under the transformation. For each rigid transformation that can match the substructures of the candidate subsegment, we test if the proteins have overlap in 3D space under such a transformation as follows:

1. Construct the grid in 3D space and mark each grid point as *interior*, *surface *or *empty *as in Step 2 with respect to each of the given proteins.

2. Let *X *be the number of grid points that are *interior *points for both proteins, *X*_1 _and *X*_2 _be the number of *interior *points of the first protein and the second protein, respectively. If *X *≤ 0.05 × *min*{*X*_1_, *X*_2_}, then we say that there is no overlap between proteins under the current rigid transformation and we output the matched substructures as the predicted binding sites.

## Results

### Comparison with existing methods

In this section, we compare our program BsFinder with three existing programs SiteEngine, SuMo, and pdbFun. They use different methods to predict the binding sites of given proteins. SiteEngine [16] is a method that recognizes the regions on the surface of one protein that are similar to the binding sites of another, and geometric hashing triangles are used to transfer the input sites into the recognized region [8]. SuMo [17] is a system for finding binding sites onto query structures, by comparing the structure of triplets of chemical groups against the binding sites found in PDB database [9]. The web server pdbFun [18] locates binding sites in proteins at the residue level, and it analyzes structural similarity between any pair of residue selections [10].

To compare BsFinder with the three existing systems, we use the proteins in PDB database, and select 55 proteins to compare with the whole database. Note that the Structural Classification of Proteins (SCOP) database [19] in [20] aims to provide a detailed and comprehensive description of the structural and evolutionary relationships between all proteins whose structures are known. It provides 11 classes to separate all known protein folds. Each class contains several different families. We choose 5 proteins from each class in different families such that there is only one entry from each family. Since BsFinder allows users to give the value of *d*, we set the threshold *d *= 1.5Å and output the matched sites with at least 15 atoms.

### Evaluation of prediction

To calculate the *precision *and *recall *value for each approach, we need to know which pair of binding sites output by the programs is real. Here we look at SitesBase [21] in [11], which holds the set of known binding sites found in PDB. The *precision *value is defined as the number of sites output by the program that are confirmed in SitesBase divided by the total number of sites output by the program, where a output site is *confirmed *in SitesBase if at least two residues of the output sites are the same as the binding sites in SitesBase. As the sites output by SuMo are very short, the sites output by SuMo are *confirmed *if each one has at least one residue which is identical to that in SitesBase. Ideally, all the sites output by the program are confirmed in SitesBase, in the case, the precision value is 100%. Apparently, the larger the precision value is, the better the program is. The *recall *value is defined as the number of sites output by the program that are confirmed in SitesBase divided by the total number of binding sites more than two complete residues for given proteins in SitesBase. If all the binding sites for given proteins in the SitesBase can be output by the program, then the recall value is 100%. Again, the larger the recall value is, the better the program is.

**Figure 3:**
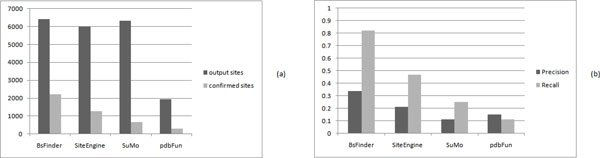
**Comparison of BsFinder, SiteEngine, SuMo, and pdbFun on 55 proteins**. (a)The average numbers of the output sites (black bar) and the confirmed sites (gray bar) for BsFinder, SiteEngine, SuMo, and pdbFun; (b)The average values of precision (black bar) and recall (gray bar) for BsFinder, SiteEngine, SuMo, and pdbFun.

We use the 55 selected proteins to compare with the whole PDB database. The results are shown in Table 1. The average numbers of the sites output by BsFinder, SiteEngine, SuMo, and pdbFun are 6425, 6003, 6329, and 1936, respectively. On average, pdbFun reports the smallest number of sites and the other three systems output approximately the same number of sites. The average numbers of the confirmed sites output by BsFinder, SiteEngine, SuMo, and pdbFun are 2218, 1265, 674, and 281, respectively. See Figure 3(a).

**Table 1:** Comparison of BsFinder, SiteEngine, SuMo, and pdbFun on 55 proteins.

	BsFinder		SiteEngine		SuMo		pdbFun	
				
	number^†^	ratio(%)^‡^	number^†^	ratio(%)^‡^	number^†^	ratio(%)^‡^	number^†^	ratio(%)^‡^
1C52	5937/1583	27/73	4798/1212	25/56	1014/159	16/8	2621/627	24/29
8GSS	6111/3243	53/95	4702/1918	41/57	4698/1603	34/48	2704/951	35/28
256B	7834/3102	40/89	3982/1410	35/41	664/100	15/3	798/221	28/7
8ICK	8984/3758	42/97	8165/1848	23/48	9398/1526	16/40	2208/372	17/10
4VHB	7122/2299	32/78	3750/855	23/30	1500/167	11/6	1014/251	25/9
2BPV	6210/1309	21/85	4689/717	15/47	842/66	8/5	1230/151	12/10
2RTO	5540/2463	44/69	3612/1350	37/38	2542/871	34/24	1744/173	10/5
2TRM	4528/1268	28/53	6984/1107	16/46	10126/919	9/38	2348/331	14/14
2XAT	6041/2783	46/72	4506/1046	23/27	4721/856	18/22	1963/230	12/6
1JJU	5074/936	18/79	5685/616	11/53	9785/381	4/33	5328/429	8/37
4FX2	5603/2667	48/68	3318/1064	32/28	1878/583	31/15	1833/439	24/12
5P21	7179/3401	47/87	6017/1998	33/52	7556/1702	23/44	2097/659	31/17
2DUB	8681/2764	32/89	7226/1734	24/57	2641/473	18/16	2673/531	20/18
3MAN	7469/3536	47/91	8974/2102	23/55	9487/1627	17/43	2371/983	42/26
6DFR	7541/3621	48/93	8054/2479	31/64	3682/877	24/23	2318/670	29/18
1J6W	8543/2361	28/88	4812/1324	28/54	2762/314	12/13	1133/276	25/11
3PYP	5733/3347	58/87	3043/932	31/25	1841/529	29/14	1713/358	21/9
1E1V	8719/2148	24/85	6704/1064	16/48	8505/882	11/39	1943/195	10/9
1OIY	8452/2981	35/92	8104/1884	23/59	8441/1121	13/35	1844/280	15/9
3BU4	1407/874	62/84	3916/948	24/38	3945/599	15/24	1089/48	4/2
1T9G	7092/2526	36/84	8927/1714	19/58	9590/1073	11/36	2243/318	14/11
7CAT	6813/1564	23/87	7241/1483	21/83	14407/875	6/49	2375/376	16/21
1JX4	5294/497	9/94	5576/314	6/60	5637/198	4/38	1843/65	4/12
1CY6	5791/477	8/95	8485/326	4/66	11855/220	2/44	2793/85	3/17
1SK6	3267/457	14/82	9713/368	4/75	17100/345	2/70	2094/79	4/16
1H2S	8437/2263	27/95	3567/967	27/41	3079/497	16/21	2912/211	8/9
1DDT	8071/1921	24/89	7446/1324	18/65	11301/904	8/45	3428/162	5/8
1U19	8186/3523	43/92	9795/2057	21/54	11298/1629	14/43	2619/508	19/13
1PPJ	6638/377	6/94	9657/332	4/83	10634/146	2/37	3509/59	2/15
1NTM	6263/916	15/87	6640/421	6/43	13164/362	3/37	3357/48	2/5
7INS	6327/3155	50/82	5827/1750	30/46	682/38	6/1	169/47	28/2
1KI0	8011/3356	42/91	7502/2014	27/55	8999/1601	18/43	876/240	27/7
1PTR	5386/1696	31/67	5503/1349	25/54	1849/296	16/12	743/94	13/4
1GMN	7970/3474	44/88	7706/2014	26/53	5955/1053	18/27	733/261	36/7
1F4L	8445/1964	23/94	8683/1459	17/71	13102/1078	8/52	3199/435	14/21
1G9B	8738/2779	32/89	5786/1959	34/68	3542/382	11/13	4354/837	19/29
1JSH	3183/1365	43/45	4251/726	17/24	8483/1246	15/41	1735/537	31/18
1MG1	8162/1220	15/91	9897/952	10/74	11225/573	5/44	2559/191	7/15
1S1C	7999/3827	48/97	7460/2382	32/62	6037/1299	22/34	1898/532	28/14
1KWX	7995/2236	28/95	6022/1765	30/75	5255/452	9/19	1831/201	11/9
1IZL	8155/3676	45/96	7196/1637	23/43	2632/477	18/15	928/244	26/7
1DWL	6558/3201	49/83	3695/1269	35/33	246/45	18/1	317/100	32/3
1FFX	6770/902	13/87	7392/422	6/41	16061/469	3/45	2699/76	3/7
3LDH	6086/2762	45/75	6013/1890	32/52	11994/1464	12/40	1924/321	17/9
2YHX	8213/2737	33/91	8279/1642	20/54	9127/1186	13/39	2250/169	8/6
1GO9	5165/2661	52/67	3571/1210	34/31	30/1	3/1	359/135	38/4
1HTH	3321/1636	49/42	2147/896	42/23	55/7	13/1	58/20	34/1
1LXF	6121/1034	17/78	4831/731	15/55	2945/145	5/11	733/25	4/2
2PRG	6147/832	14/86	6298/718	12/74	6151/190	3/20	1873/71	4/8
1H2K	964/43	4/94	2779/26	2/58	8038/108	1/40	2017/18	1/40
1G8X	6989/3605	52/91	8935/2029	23/54	16085/2318	14/62	5733/797	14/21
1JY4	4207/2171	52/56	2678/756	28/20	129/35	27/1	105/49	5/1
1K09	3123/638	20/82	1201/291	24/38	123/4	3/1	97/16	16/2
1ABZ	2825/2432	86/63	1354/717	53/19	44/1	2/1	255/93	36/3
1L6X	8142/3627	45/96	7036/2059	29/55	5128/1005	20/27	858/355	41/10

^†^The first number is the number of output sites reported by the program, the second number is the number of confirmed sites reported by the program;

^‡^The first number is the precision value (%) for the program, the second number is the recall value (%) for the program;

The precision and recall values for 55 proteins output by four programs are shown in Table 1. Apparently, BsFinder has the largest precision and recall values for most of the cases. On average, the precision value of BsFinder is 34% while the precision values for SiteEngine, SuMo, and pdbFun are 21%, 11%, and 15%, respectively. The average recall value of BsFinder is 82% while the average recall values for SiteEngine, SuMo, and pdbFun are 47%, 25%, and 11%, respectively. See Figure 3(b). The value of recall is very important in practice. From the experiment results, we know that the existing programs have lower values of recall.

The possible reasons that our method can get better results might be (1) we use the surface information, (2) we look at the similarity of two local 3D substructures in terms of rigid transformation while the previous methods use triples of atoms or pairs of amino acids and (3) the volumes of the protein molecules are considered when the rigid transformation is computed.

### Comparison of running time

To compare the running time of different programs, we use a Pentium(R) 4 (CPU of 2.40 GHz) to run all four programs. Based on 55 selected proteins, the average running times of BsFinder, SiteEngine, SuMo, and pdbFun for comparing each given protein with the whole PDB database are roughly 50 minutes, 70 minutes, 30 minutes, and 5 minutes, respectively. See Table 2. Thus, BsFinder is the second slowest program. However, it is still faster than SiteEngine which has the highest average values of precision and recall among the three existing programs.

**Table 2:** Comparison of four programs.

	Running Time	Precision	Recall
BsFinder	50 minutes	34%	82%
SiteEngine	70 minutes	21%	47%
SuMo	30 minutes	11%	25%
pdbFun	5 minutes	15%	11%

### Performance of programs for different families

**Figure 4:**
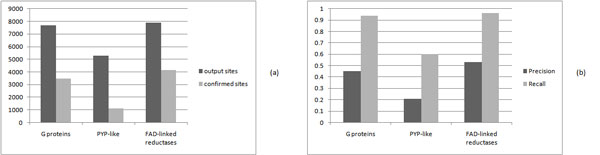
**Results of BsFinder on three different families**. (a)The average numbers of the output sites (black bar) and the confirmed sites (gray bar) for three different families; (b)The average values of precision (black bar) and recall (gray bar) for three different families.

To see the performance of programs for different protein families, we look at three different families (G proteins family in P-loop folds, PYP-like family in Profilin-like folds, and FAD-linked reductases family in FAD/NAD(P)-binding folds) and select five proteins from each family. The average numbers of matched sites output by BsFinder for three families are 7680, 5289, and 7892, respectively. The average numbers of confirmed sites for three families are 3487, 1132, and 4138, respectively. The average precision values for three families are 45%, 21% and 53%, respectively. The average recall values for three families are 94%, 60% and 96%, respectively. The results are shown in Figure 4.

#### G proteins family in P-loop folds

We select 5 proteins (1A2B, 1CXZ, 1DPF, 1FTN, 1S1C) from G proteins family in P-loop folds. The results are shown in Table 3. The precision values of BsFinder (48%, 46%, 43%, 42% and 47%) are larger than those of other three programs. The recall values of BsFinder (95%, 93%, 92%, 91% and 99%) are more than 90%, while the recall values of the other three programs are almost less than 40%.

**Table 3:** Comparison of the four programs on G proteins family in P-loop folds.

	BsFinder		SiteEngine		SuMo		pdbFun	
				
	number^†^	ratio(%)^‡^	number^†^	ratio(%)^‡^	number^†^	ratio(%)^‡^	number^†^	ratio(%)^‡^
1A2B	7601/3647	48/95	6579/1717	26/40	6375/1209	19/29	1787/381	21/9
1CXZ	7832/3602	46/93	7425/1480	20/35	8388/1433	18/34	2696/403	15/10
1DPF	7537/3241	43/92	5975/1343	23/34	4702/1029	22/26	1993/365	18/10
1FTN	7435/3121	42/91	7147/1471	21/35	8599/1328	16/31	2232/414	19/11
1S1C	7995/3827	47/99	7460/1382	19/36	6037/1299	22/34	1898/532	28/14

^†^The first number is the number of output sites reported by the program, the second number is the number of confirmed sites reported by the program;

^‡^The first number is the precision value (%) for the program, the second number is the recall value (%) for the program;

#### PYP-like family in Profilin-like folds

We select 5 proteins (1D7E, 1F9I, 1KOU, 1NWZ, 2PHY) from PYP-like family in Profilin-like folds. The results are shown in Table 4. The precision values of BsFinder (17%, 18%, 24%, 25% and 21%) are similar to those of the other three programs. The recall values of BsFinder (58%, 64%, 59%, 63% and 57%) are larger than that of the other three programs.

**Table 4:** Comparison of the four programs on PYP-like family in Profilin-like folds.

	BsFinder		SiteEngine		SuMo		pdbFun	
				
	number^†^	ratio(%)^‡^	number^†^	ratio(%)^‡^	number^†^	ratio(%)^‡^	number^†^	ratio(%)^‡^
1D7E	4845/834	17/58	5017/698	14/48	2582/173	7/13	223/33	15/3
1F9I	5771/1068	18/64	5680/740	13/44	3405/224	7/14	203/13	7/1
1KOU	5352/1297	24/59	4521/896	20/41	2421/264	11/13	916/80	9/4
1NWZ	5027/1279	25/63	5497/914	17/45	2096/243	12/13	206/23	12/2
2PHY	5451/1189	21/57	4014/821	20/39	3178/285	9/14	208/15	8/1

^†^The first number is the number of output sites reported by the program, the second number is the number of confirmed sites reported by the program;

^‡^The first number is the precision value (%) for the program, the second number is the recall value (%) for the program;

#### FAD-linked reductases family in FAD/NAD(P)-binding folds

We select 5 proteins (1B4V, 1B8S, 1COY, 1IJH, 3COX) from FAD-linked reductases family in FAD/NAD(P)-binding folds. The results are shown in Table 5. The precision values of BsFinder (54%, 52%, 53%, 53% and 54%) are all more than 50%. The recall values of BsFinder (97%, 96%, 96%, 96% and 98%) are very close to 100%.

**Table 5:** Comparison of the four programs on FAD-linked reductases family in FAD/NAD(P)-binding folds.

	BsFinder		SiteEngine		SuMo		pdbFun	
				
	number^†^	ratio(%)^‡^	number^†^	ratio(%)^‡^	number^†^	ratio(%)^‡^	number^†^	ratio(%)^‡^
1B4V	7835/4138	54/97	7017/1998	29/47	11797/1925	17/46	6894/2381	34/56
1B8S	7996/4101	52/96	7680/1940	26/46	11929/1865	16/44	9408/2843	31/68
1COY	7892/4135	53/96	8521/1996	24/47	11989/1864	16/43	9407/2857	31/67
1IJH	7859/4119	53/96	8497/2014	24/47	11427/1894	17/45	9424/2662	29/63
3COX	7878/4199	54/98	8014/2021	26/48	11647/1775	16/42	9245/2850	31/67

^†^The first number is the number of output sites reported by the program, the second number is the number of confirmed sites reported by the program;

^‡^The first number is the precision value (%) for the program, the second number is the recall value (%) for the program;

**Figure 5:**
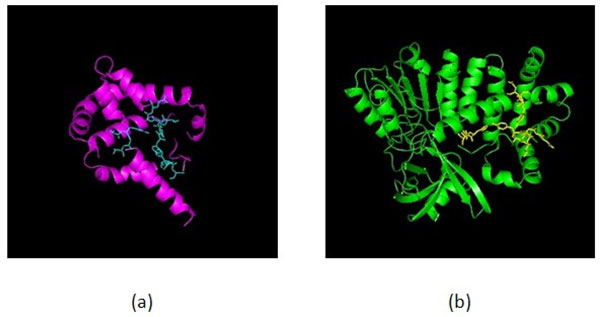
**The 3D structures of proteins 4VHBA (a) and 1CQXA (b)**.

**Figure 6:**
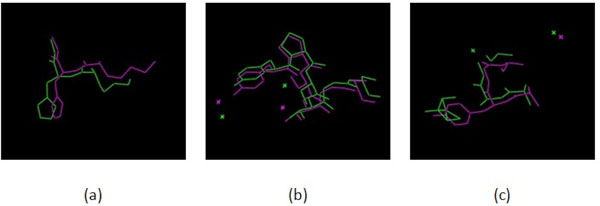
**The similar sites for 4VHBA and 1CQXA predicted by BsFinder**. (a)residues 84-86 from 4VHBA and residues 84-86 from 1CQXA; (b) residues 95-100 from 4VHBA and residues 95-100 from 1CQXA; (c) residues 125-128 from 4VHBA and residues 125-128 from 1CQXA.

**A Case: **We compare two proteins 4VHBA and 1CQXA. The cartoon version of the protein 3D structures are shown in Figure 5, and the matched parts of structures are shown as the sticks fashion. BsFinder finds a rigid transformation that matches residues 84-86 from 4VHBA to residues 84-86 from 1CQXA, residues 95-100 from 4VHBA to residues 95-100 from 1CQXA, and residues 125-128 from 4VHBA to residues 125-128 from 1CQXA. See Figure 6. The three pairs of matched sites are confirmed in SitesBase. Note that these three pairs can be matched under one rigid transformation simultaneously.

### Searching similar binding sites

BsFinder can use a binding site to search the similar sites in the protein structures database. SiteEngine can search a given functional site on a large set of complete protein structures. SuMo can search for the given 3D site of interest among the structures of the PDB. PAST [22] is a web service based on an adaptation of the generalized suffix tree and relies on a linear representation of the protein backbone [23]. PAST can find the functional sites from the protein structures database similar to the given binding site.

**Figure 7:**
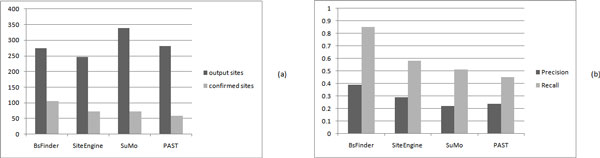
**Comparison of BsFinder, SiteEngine, SuMo, and PAST on 100 sites**. (a)The average numbers of the output sites (black bar) and the confirmed sites (gray bar) for BsFinder, SiteEngine, SuMo, and PAST; (b)The average values of precision (black bar) and recall (gray bar) for BsFinder, SiteEngine, SuMo, and PAST.

We randomly select the 100 binding sites with different types from the SitesBase and search the whole PDB database. The average numbers of the sites output by BsFinder, SiteEngine, SuMo, and PAST are 274, 266, 399, and 281, respectively. The average numbers of the confirmed sites output by BsFinder, SiteEngine, SuMo, and PAST are 106, 73, 72, and 58, respectively. See Figure 7(a). BsFinder finds a relatively smallest number of output sites, and the number of confirmed sites output by BsFinder is the biggest. Apparently, BsFinder has the largest precision and recall values for most of the cases. On average, the precision value of BsFinder is 39% while the precision values for SiteEngine, SuMo, and PAST are 27%, 22%, and 24%, respectively. The average recall value of BsFinder is 86% while the average recall values for SiteEngine, SuMo, and PAST are 58%, 51%, and 45%, respectively. See Figure 7(b).

## Discussion

### The gaps in binding sites

In the first step of our algorithm, we do sequence alignment where each letter is an atom. This allows the matched sites to have some missed atoms, and each missed atom represents one gap in the binding sites. Step 1 is very important for predicting binding sites on proteins. Among the output sites, 67127 of them do not contain any gap, 63593 contain one gap, 77725 contain two gaps, 81259 contain three gaps, 38863 contain four gaps, 21198 contain five gaps and 3533 contain more than five gaps. The gap distribution of the confirmed sites are 18285 (no gap), 19504 (one gap), 26809 (two gaps), 26809 (three gaps), 15847 (four gaps), 12197 (five gaps) and 2452 (more than five gaps). The confirmed sites have higher proportion of the four or more gaps among all output sites reported by BsFinder.

### The power of surface detection

In Step 2 of our algorithm, we identify the surface atoms in the given proteins and rule out the substructures in which less than 2/3 of atoms are the surface atoms for further calculation of the rigid transformation. To demonstrate the effect of Step 2, we compare the final version of BsFinder with the version without Step 2. By adjusting the parameters, the final version of BsFinder has improved precision value while the recall value remains essentially unchanged. The average precision values for BsFinder without Step 2 and the final version of BsFinder are 29% and 34%, respectively. The average recall values for BsFinder without Step 2 and the final version of BsFinder are 83% and 82%, respectively. Therefore, by doing Step 2 the precision value can be improved by about 5%. This is a significant improvement.

## Conclusions

We have developed a program for finding binding sites on the given proteins. Our method uses the 3D structure information to detect the similar surface regions. Experiments show that our program outperforms all existing programs.

## Competing interests

The authors declare that they have no competing interests.

## Authors' contributions

FG is in charge of the software package development and experiment design. LW is in charge of algorithm design and manuscript preparation. All authors read and approved the final manuscript.
